# Population Genetic Structure of *Aedes fluviatilis* (Diptera: Culicidae)

**DOI:** 10.1371/journal.pone.0162328

**Published:** 2016-09-06

**Authors:** Laura Cristina Multini, André Barretto Bruno Wilke, Lincoln Suesdek, Mauro Toledo Marrelli

**Affiliations:** 1 Departamento de Epidemiologia, Faculdade de Saúde Pública, Universidade de São Paulo, São Paulo, SP, Brasil; 2 Instituto de Medicina Tropical de São Paulo, Universidade de São Paulo, São Paulo, SP, Brasil; 3 Laboratório de Parasitologia, Instituto Butantan, São Paulo, SP, Brasil; University of Missouri Columbia, UNITED STATES

## Abstract

Although *Aedes fluviatilis* is an anthropophilic mosquito found abundantly in urban environments, its biology, epidemiological potential and genetic characteristics are poorly understood. Climate change and urbanization processes that result in environmental modifications benefit certain anthropophilic mosquito species such as *Ae*. *fluviatilis*, greatly increasing their abundance in urban areas. To gain a better understanding of whether urbanization processes modulate the genetic structure of this species in the city of São Paulo, we used eight microsatellite loci to genetically characterize *Ae*. *fluviatilis* populations collected in nine urban parks in the city of São Paulo. Our results show that there is high gene flow among the populations of this species, heterozygosity deficiency and low genetic structure and that the species may have undergone a recent population expansion. There are two main hypotheses to explain these findings: (i) *Ae*. *fluviatilis* populations have undergone a population expansion as a result of urbanization; and (ii) as urbanization of the city of São Paulo occurred recently and was quite intense, the structuring of these populations cannot be observed yet, apart from in the populations of Ibirapuera and Piqueri parks, where the first signs of structuring have appeared. We believe that the expansion found in *Ae*. *fluviatilis* populations is probably correlated with the unplanned urbanization of the city of São Paulo, which transformed green areas into urbanized areas, as well as the increasing population density in the city.

## Introduction

Urbanization is often a chaotic process that causes environmental stress, leading to the domiciliation of insects that have adapted to man-made changes [1–3]. Culicids are an example of such insects and can be found abundantly in metropolitan areas, where the environment can favor a few species that have adapted to it. These species can be not only a source of nuisance but also in some cases potential disease vectors (e.g., *Aedes aegypti*, *Aedes albopictus*, *Culex quinquefasciatus*, *Culex nigripalpus*, *Aedes scapularis* and *Aedes fluviatilis*) [1,2,4–9].

*Ae*. *fluviatilis* (Lutz, 1904) is a mosquito species native of Brazil found abundantly in urban areas. It is distributed in the Neotropics and can be found from Mexico to Argentina. The species is able to survive in wild, semi-wild, suburban and urban areas [10,11]. Indeed, it successfully inhabits urban environments and is found abundantly in the city of São Paulo, where it represented around 10% of all mosquito specimens collected in recent studies, indicating that it is well adapted and established in this urban environment [7,12]. Females of the species are highly anthropophilic and have been reported to ingest human blood while their eggs are developing [13]. However, as there are few studies on *Ae*. *fluviatilis* in the literature, it is considered a neglected species.

*Ae*. *fluviatilis* is considered a potential vector of yellow fever virus [14], *Plasmodium gallinaceum* and *Dirofilaria immitis* [15–17], and although it is naturally infected with *Wolbachia* (w*Flu*), this causes only incomplete cytoplasmic incompatibility and has no effect on its infection by *P*. *gallinaceum* [18,19].

The success of some species of mosquitoes in inhabiting urban environments and the consequent increase in their abundance can be attributed to two factors: the availability of breeding sites and the availability of blood-meal sources [20]. Along with climate changes, these factors can modulate the size of mosquito populations in urban areas [21–23]. Therefore, a better knowledge of the genetic structure of urban mosquitoes can lead to a better understanding of how *Ae*. *fluviatilis* populations are modulated by selective pressures in the environment. Microsatellites can be used in genetic population studies as they are highly polymorphic and assumed to be neutral markers. They flank conserved regions of the genome and amplify polymorphic regions [24]. Hence, as they are believed not to be subjected to selective pressures, microsatellites can be a valuable tool for population genetic studies of mosquitoes on a macrogeographic [23,25,26] and microgeographic [27,28] scale.

Genetic structure studies of vector insect species conducted on a microgeographic scale found structuring in sympatric *Triatoma infestans* populations in Argentina [28], and Olanratmanee et al. [27] found genetic structuring in *Ae*. *aegypti* populations in villages no more than 10 km apart in Thailand. In the latter case, the structuring may have been caused by genetic drift due to adult mosquito oviposition patterns. These studies proved the effectiveness of microsatellite markers in identifying fine-scale genetic differentiation.

In light of the above, this study used microsatellite markers to investigate how *Ae*. *fluviatilis* populations are genetically structured in the city of São Paulo and whether urbanization processes can modulate the genetic structure of this culicid.

## Material and Methods

### Specimen collection

**Fig 1:**
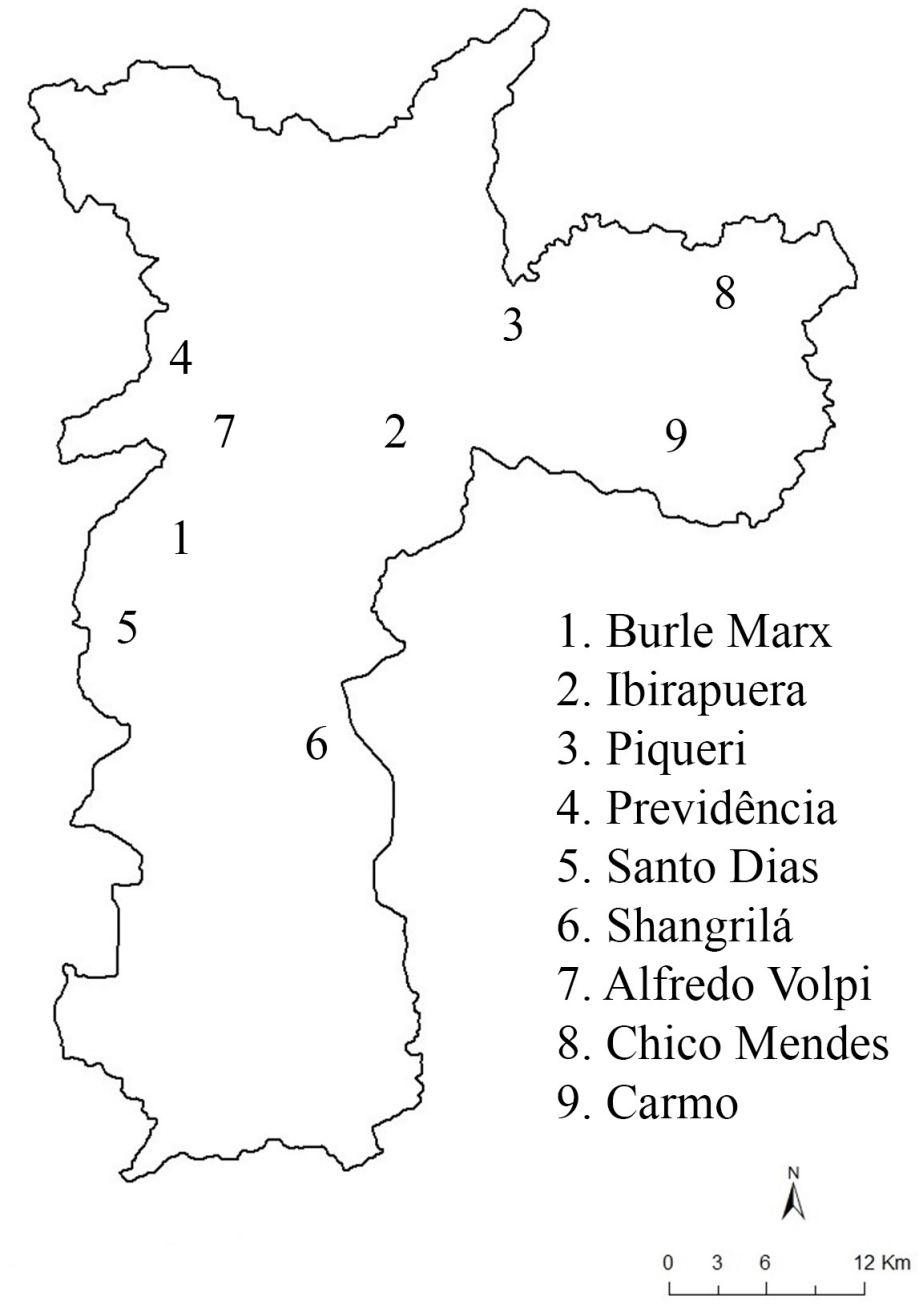
*Aedes fluviatilis* sampling locations in the city of São Paulo.

*Ae*. *fluviatilis* mosquitoes were collected from nine urban parks (Burle Marx, Ibirapuera, Piqueri, Previdência, Santo Dias, Shangrilá, Alfredo Volpi, Chico Mendes and Carmo) in different areas of the city of São Paulo, Brazil (Fig 1). Mosquito collections were performed monthly from March 2011 to February 2012 and from August 2012 to July 2013. Adult mosquitoes were collected with portable, battery-powered aspirators [29] and CDC CO_2_-baited light traps [30] (Table 1). The study was approved by the Ethical Committee of the University of São Paulo, and collection permits were provided by the Department of the Environment and Green Areas.

**Table 1:** *Aedes fluviatilis* sampling information.

Population	Coordinates	Number of *Ae*. *fluviatilis* collected (%*)	Females used	Collection year
Burle Marx	23°37’54”S 46°43’16”W	1,264 (28.5%)	30	2012/2013
Ibirapuera	23°35’14”S 46°39’27”W	247 (9.5%)	30	2011/2012
Piqueri	23°31’40”S 46°34’14”W	1,104 (12%)	30	2012/2013
Previdência	23°34’49”S 46°43’33”W	157 (7%)	30	2012/2013
Santo Dias	23°39’50”S 46°46’23”W	231 (10%)	30	2011/2012
Shangrilá	23°45’41”S 46°40’06”W	1,006 (15%)	30	2011/2012
Alfredo Volpi	23°35’16”S 46°42’09”W	151 (12.5%)	30	2011/2012
Chico Mendes	23°30’24”S 46°25’44”W	151 (3%)	30	2011/2012
Carmo	23°35’04”S 46°27’47”W	76 (7%)	30	2011/2012

*Number of *Aedes fluviatilis* specimens as a percentage of the total number of mosquitoes collected (Medeiros-Sousa et al., unpublished data).

### DNA extraction and PCR reactions

DNAs were extracted using the DNEasy Blood and Tissue Kit (Qiagen, Hilden, Germany) following the manufacturer’s protocol. PCR reactions were carried out with eight microsatellite primers originally designed for *Ae*. *aegypti*, *Ae*. *albopictus* and *Ae*. *caspius* and successfully used with *Ae*. *fluviatilis* by Multini et al. [31]. The primers were labeled with a fluorescent dye (FAM, HEX or NED), and the PCR reactions were performed as in Porretta et al. [32], Chambers et al. [33] and Beebe et al. [34] in an E6331000025 Eppendorf Thermocycler (Mastercycler Nexus Gradient, Eppendorf, Hamburg, Germany). The amplified fragments were visualized on a 1% agarose gel stained with GelRed^™^ (Biotium, Hayward, CA, USA) and examined under UV light.

PCR products were diluted 1:7 by mixing 3 μL of each product labeled with a different dye with 21 μL of Ultra-Pure Water (Applied Biosystems, Foster City, CA, USA) to a final volume of 30 μL. A second dilution was performed with 2 μL of the previous dilution suspended in 8.925 μL of Hi-Di formamide (Applied Biosystems, Foster City, CA, USA) and 0.075 μL of GeneScan 500 ROX size standard (Applied Biosystems, Foster City, CA, USA) to a final volume of 11 μL. The samples were then sent to the University of São Paulo Center for Human Genome Studies and size-sorted in an ABI 3730 automatic sequencer (Applied Biosystems, Foster City, CA, USA). Fragment analysis was performed with Gene Marker (v1.85 SoftGenetics, State College, PA, USA).

### Genetic Analysis

Allele frequency, observed heterozygosity (H_O_), expected heterozygosity (H_E_), deviations from Hardy-Weinberg equilibrium (*P*-values were adjusted using Bonferroni correction), inbreeding coefficient (F_IS_), linkage disequilibrium and gene flow were calculated using Genepop (v4.2 http://genepop.curtin.edu.au/) and Arlequin (v3.5) [35,36]. Allelic richness and private allelic richness were calculated using HP-Rare (v1.0) [37].

The probability of null alleles occurring was calculated for each locus for each population using FreeNa [38]. The same software was also used to estimate genetic heterogeneity (F_ST_) and Cavalli-Sforza and Edwards chord distance taking into account the presence of null alleles. To compute pairwise F_ST_ values and their significance for all the populations, Arlequin (v3.5) [39] with 50,000 permutations was used

Linear correlation analyses between F_ST_/(1-F_ST_) and geographic distance (km) and between F_ST_/(1-F_ST_) and environmental variables such as elevation, slope (variation in elevation vs. area), perimeter-to-area ratio (Patton index) [39], green area per inhabitant (m^2^) in the vicinity of the park, monthly accumulated rainfall, mean annual temperature and area of the park (km^2^) [25,40] were performed using PAST (v3.11) [41]. A dendrogram displaying the Cavalli-Sforza and Edwards chord distance was constructed using Statistica 7.0 [42].

The amplified alleles were subjected to Bayesian model-based clustering analysis using Structure (v2.3.3) [43]. The estimated number of clusters k (ΔK), which identifies genetically homogeneous groups of individuals, was calculated with Structure Harvester (Web v0.6.94) [44]. To identify genetic drift, an analysis to determine whether the loci show heterozygosity deficiency or excess was performed in Bottleneck (v1.2.2). This analysis compares two heterozygosity scenarios: (i) the expected heterozygosity based on allele frequencies (He) and (ii) the expected heterozygosity based on observed alleles (Heq). He > Heq therefore indicates a recent Bottleneck event and He < Heq a recent population expansion [45].

## Results

### Marker assessment

Hardy-Weinberg equilibrium tests were conducted for all eight microsatellite loci for each locus and population. H_O_ was higher than H_E_ in 37 of the 64 tests, and the average F_IS_ was 0.142 (S1 Table). After 150 possible tests for linkage disequilibrium, none of the linkage results could be considered statistically significant since no two loci were linked more than once across the tested populations [31]. Allelic richness ranged from 3.4 (Ibirapuera) to 4.72 (Carmo), and private allelic richness was moderate, ranging from 0.13 (Ibirapuera) to 1.19 (Carmo) (S2 Table).

The tests to estimate the probability of null alleles showed that this was high for the Albtri3 locus in five populations (Burle Marx, Previdência, Alfredo Volpi, Chico Mendes and Carmo), for the OchcB5 locus in four populations (Ibirapuera, Piqueri, Santo Dias and Alfredo Volpi), for the OchcB9 locus in two (Burle Marx and Chico Mendes) and for the Albtri33 locus in one (Ibirapuera). The probability of null alleles in the populations studied ranged from 0 to 0.19 for the Albtri3 locus, 0 to 0.28 for OchcB5, 0 to 0.18 for OchcB9 and 0 to 0.16 for Albtri33. The OchcD11, Albtri20, Albtri44 and AEDC loci had low (<0.07) or zero probabilities of null alleles.

### Genetic differentiation

F_ST_ values ranged from 0 to 0.02, indicating low genetic structure among the populations; 67% of these values were statistically significant. The F_ST_ values calculated by FreeNA (which corrects for the bias induced by the presence of null alleles) ranged from 0.0002 to 0.05, indicating low to moderate genetic structure. There was therefore no statistically significant difference between the corrected and uncorrected values of F_ST_ (Table 2). Gene flow among the populations after correction for sample size was 7.74 per generation per population, indicating a high degree of allelic similarity among these populations.

**Table 2:** Pairwise F_ST_* estimates for *Aedes fluviatilis* populations.

**Population**	**Burle Marx**	**Ibirapuera**	**Piqueri**	**Previdência**	**Santo Dias**	**Shangrilá**	**Alfredo Volpi**	**Chico Mendes**	**Carmo**
**Burle Marx**	-	0.050189	0.040068	0.019847	0.031485	0.026849	0.027445	0.008033	0.007975
**Ibirapuera**	**0.02532**	-	0.000709	0.028792	0.029321	0.035701	0.034906	0.045820	0.040354
**Piqueri**	**0.01921**	0	-	0.016828	0.023858	0.025561	0.020121	0.025722	0.024515
**Previdência**	**0.00689**	**0.01076**	0	-	0.006230	0.006344	0.016797	0.006850	0.001337
**Santo Dias**	**0.02340**	**0.02643**	**0.01208**	0.00016	-	0.009239	0.025782	0.014845	0.013426
**Shangrilá**	**0.02427**	**0.02051**	**0.00764**	0.00111	**0.00375**	-	0.024413	0.018144	0.015420
**Alfredo Volpi**	**0.01683**	**0.01821**	**0.00416**	0.00263	**0.01906**	**0.01352**	-	0.009282	0.011423
**Chico Mendes**	0.00295	**0.02415**	**0.00687**	0	**0.00811**	**0.01704**	0.00208	-	0.000215
**Carmo**	0	**0.01780**	**0.00581**	0	**0.00528**	**0.00878**	0.00075	0	-

*Below the diagonal: F_ST_ values without correction for null alleles. Significant values are in bold. Above the diagonal: FreeNA corrected F_ST_ values.

### Genetic distance

**Fig 2:**
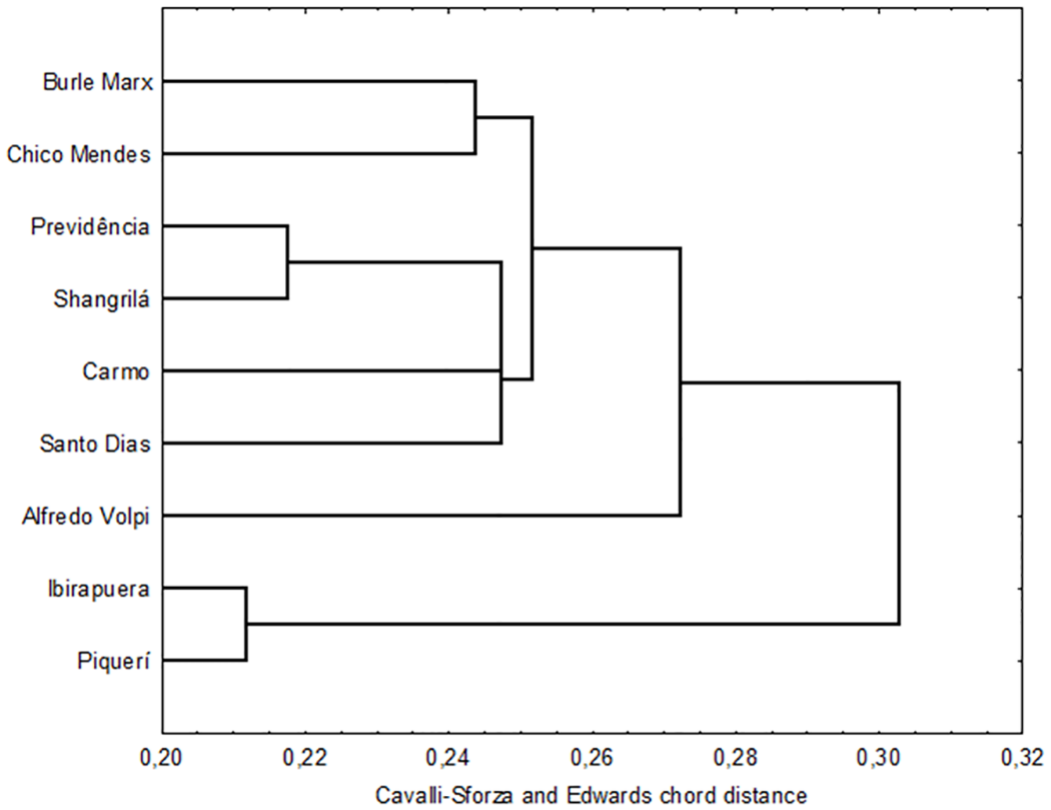
Genetic-distance dendrogram for *Aedes fluviatilis*.

The dendrogram constructed using the Cavalli-Sforza and Edwards chord distance was not consistent with the geographic distances between populations and showed two main clusters, one (a) containing the populations from Ibirapuera and Piqueri, which segregated together and are fairly different from the other populations, and another (b) with the remaining populations. Cluster (b) segregated into two subclusters, one containing similar populations with no great differences between them (Burle Marx, Chico Mendes, Previdência, Shangrilá, Carmo and Santo Dias), and the other formed by the population from Alfredo Volpi (Fig 2).

Linear correlation analysis of geographic and genetic distance showed that there is no correlation between these two variables in the populations tested (r = -0.2421; r^2^ = 0.058614; *P* = 0.15485), and a similar analysis of genetic distance and environment variables indicated that the former does not correlate with any of the variables tested apart from slope (r = -0.6694; r^2^ = 0.4481; *P* = 0.048595) (S3 Table).

### Bayesian cluster analysis

**Fig 3:**
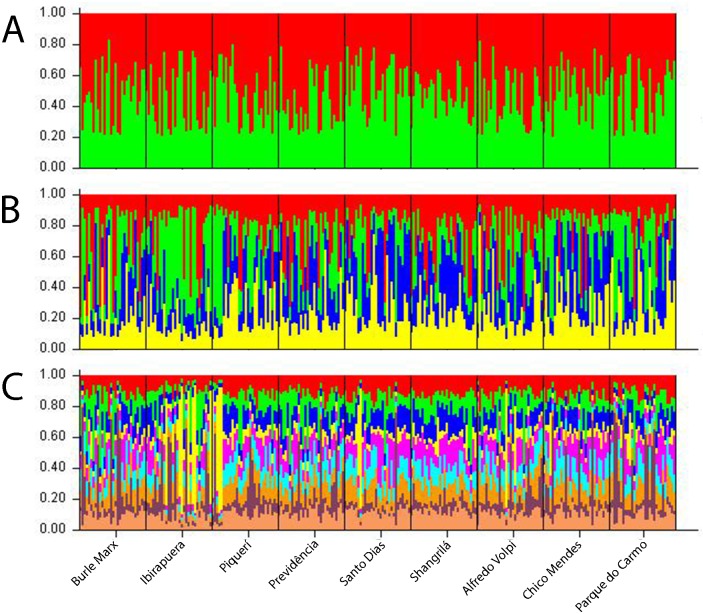
Bayesian analysis of structure for all *Aedes fluviatilis* populations showing the subdivision of individuals k = 2 (A), k = 4 (B) and k = 9 (C). Each of the 270 individuals from nine populations is represented by a vertical line divided into different colored segments. The length of each segment represents the probability of the individual belonging to the genetic cluster represented by that color.

The results of the Bayesian analysis and application of Evanno’s method were used to identify the k value that represents the number of groups that best explains the results [44], which was 2 (S1 Fig), indicating that the populations can be divided into two genetic groups, albeit without any visible differences between them (Fig 3A). Subsequently, other k values were tested; k = 4 indicated that the Ibirapuera and Piqueri populations are structured in a distinct genetic group, reflected in the higher prevalence of the color green; the Santo Dias and Shangrilá populations are also structured, but with a higher prevalence of the color blue (Fig 3B). Finally, k = 9 was tested and the results indicated that all the populations have a homogenous genetic pattern apart from those in Ibirapuera and Piqueri, which are structured as a distinct genetic group from the remaining populations and have a higher prevalence of the color yellow (Fig 3C).

### Population Dynamics

While the heterozygosity tests conducted in Bottleneck under the SMM (Stepwise Mutation Model) showed more loci with heterozygosity deficiency in eight of the nine populations, *P*-values were only significant (<0.05) for the Piqueri and Carmo populations and were borderline for the Burle Marx and Alfredo Volpi populations (Table 3). These results suggest that the *Ae*. *fluviatilis* populations have probably suffered a population expansion (*He < Heq*).

**Table 3:** *Aedes fluviatilis* heterozygosity tests.

		Burle Marx	Ibirapuera	Piqueri	Previdência	Santo Dias	Shangrilá	Alfredo Volpi	Chico Mendes	Carmo
**SMM**	*He < Heq*	4	4	7	4	4	2	5	4	5
	*He > Heq*	2	3	1	3	4	5	2	3	2
	*P (He < Heq)*	0.07813	0.28906	**0.00977**	0.28906	0.23047	0.59375	0.05469	0.23438	**0.03906**

Number of loci exhibiting heterozygosity excess (*He*) and expected heterozygosity based on the number of observed alleles (*Heq*) under the SMM model. Significant *P*-values for heterozygosity deficiency are in bold.

In a subsequent analysis in which all 270 specimens were considered one population, the results showed six loci with heterozygosity deficiency (*H*_*e*_ *< H*_*eq*_) and a significant *P*-value (*P* = 0.01367), lending support to our finding that the populations in this study have probably suffered a recent population expansion.

## Discussion

Mosquito species that can survive in urban environments have a significant advantage over sylvatic species because there tends to be less larval competition and species richness in these environments as well as fewer natural predators and greater availability of breeding sites and hosts for blood feeding, leading to an increase in the population of these species and the territory they occupy [46].

Developing countries frequently experience rapid, unplanned urbanization, which is characterized by a lack of basic sanitation, unsanitary houses, polluted rivers and untreated sewage. These factors favor an increase in the abundance of mosquito species that have adapted to urban environments and become an obstacle to effective vector control strategies [1,2]. Furthermore, urbanization processes tend to alter the micro-climate, which, together with global warming, may increase the abundance of mosquitoes in large cities [2]. Models using African malaria vectors and weather data suggest that a 0.5% increase in average temperature may result in a 30–100% increase in mosquito abundance [47].

Our findings suggest that *Ae*. *fluviatilis* populations in the city of São Paulo have undergone a population expansion. This conclusion is supported by the low genetic structure, high gene flow and heterozygosity deficiency found in this study. The results also suggest that this population expansion may be occurring in parallel with the growth of the city. Expansion of mosquito populations has previously been reported for several species of mosquitoes (e.g., *An*. *gambiae*, *An*. *arabiensis*, *An*. *darlingi* and *An*. *funestus*). In the case of these mosquitoes, the same phenomena were observed, namely, recent population expansion, high gene flow and an absence of isolation by distance (IBD) [48–50], suggesting that population expansion is a common trend among mosquito populations that can cope with man-made alterations and human expansion.

There are two main hypotheses to explain the low genetic structure in the *Ae*. *fluviatilis* populations studied here: (i) *Ae*. *fluviatilis* population expansion happened because this species is well adapted to the urban environment and is able to complete its entire life cycle within the city. Therefore, urbanization may have been beneficial to this species and a major factor contributing to population expansion, a hypothesis supported by the finding of high gene flow in these populations, which indicates a homogenizing genetic effect [51]. This phenomenon has previously been observed in *Ae*. *taeniorhynchus* mosquito populations from Colombia [52,53].

Bayesian analysis revealed low genetic structure among the populations studied, although the Ibirapuera and Piqueri populations, which are quite similar to each other, are different from the other populations. This segregation can also be observed in the dendrogram. These parks, which are located in densely populated areas, have undergone major environmental changes as a result of human activities and urbanization. More than 1 million people visit Ibirapuera park every month, and Piqueri park is located next to a major highway, where more than 400 thousand vehicles circulate every day [7,12].

These results indicate that genetic structuring may be occurring in the Ibirapuera and Piqueri populations, which leads to the second hypothesis: (ii) the city of São Paulo’s recent history of intense, haphazard urbanization starting in the 1960s [54] suggests that the *Ae*. *fluviatilis* population was actually a large population that became fragmented as the city grew. As this urbanization occurred recently and was very intense, structuring in these populations cannot be observed yet apart from in the Ibirapuera and Piqueri populations, where the first signs of population structuring are apparent.

The absence of IBD indicates that urbanization has a greater influence on population structure than distance does. Similar results were also found for populations of other *Aedes* species (e.g., *Ae*. *aegypti* and *Ae*. *japonicus*), in which the authors did not observe IBD on either a microgeographic or macrogeographic scale [27,55,56], suggesting that the species of this genus tend to exhibit genetic similarities. Despite the absence of environmental correlation in the present study, it is known that mosquitoes are ectothermic organisms and that their growth, survival and behavior are strongly related to environmental conditions [57,58]. Other variables may therefore be influencing population expansion in *Ae*. *fluviatilis*, including environmental changes caused by humans in urban areas [58].

The Hardy-Weinberg equilibrium tests for each locus and each population indicating heterozygosity excess in the present study may be due to binomial sampling error [59], as when the same test was conducted by Multini et al. [31], who considered all individuals to be one population, heterozygosity deficiency was observed. This latter finding would be expected in the present study, as the *Ae*. *fluviatilis* populations studied here show low genetic structure, a common trait in *Aedes* species [27,60].

The allelic richness found in *Ae*. *fluviatilis* in this study was similar to that of other *Aedes* species [60,61], indicating that the results represent the genuine allelic richness and are not the result of limitations of the loci. The private allelic richness found in the populations was moderate, suggesting some degree of isolation among the populations, although this may be related to the fact that the microsatellite primers used were originally designed for other species of *Aedes*, which could also explain the presence of null alleles [31,62,63].

Secondary mosquito vectors are commonly neglected in genetic population studies. However, although *Ae*. *fluviatilis* is not directly implicated in the transmission of pathogens to humans, it can be found in large numbers in urban areas. Its epidemiological role has yet to be elucidated, and infectivity studies on this mosquito are dated; the yellow fever virus infectivity test was carried out in 1931 [14] and, to our knowledge, there are no studies on the infectivity of *Ae*. *fluviatilis* by the dengue, chikungunya and Zika viruses. In addition, there is an inherent risk of new pathogens being introduced, especially in large, populous cities [64,65]. This scenario was observed with the mosquito *Cx*. *tarsalis* in the United States and the arrival of West Nile virus [66] and also with *Ae*. *ochraceus*, which acted as an important vector in the epidemic caused by Rift Valley fever virus in Kenya in 2007 [67].

The *Ae*. *fluviatilis* population structure patterns found in this study revealed a significant population expansion that we believe to be associated not only with the transformation of green areas into urbanized areas, but also with the increasing human population density in large cities, representing a scenario in which there is an ever-greater risk of disease transmission and epidemics.

## Supporting Information

S1 Fig Graph of ΔK showing k = 2 as the most probable number of genetic groups. (TIF)

S1 Table Analysis of the genetic diversity of *Aedes fluviatilis* using eight microsatellite loci. Significant *P*-values in bold.
(DOCX)

S2 Table Allele frequencies for the eight loci analyzed in *Aedes fluviatilis* populations. Allelic richness (Na) and private allelic richness (Np).
(DOCX)

S3 Table Linear correlation analysis of genetic distance [F_ST_/(1-F_ST_)] and environmental variables. *Significant *P*-value.
(DOCX)

## References

[1] Taipe-Lagos CB, Natal D. Culicidae mosquito abundance in a preserved metropolitan area and its epidemiological implications. Rev Saude Publica. 2003;37: 275–279. 10.1590/S0034-89102003000300002 12792675

[2] Li Y, Kamara F, Zhou G, Puthiyakunnon S, Li C, Liu Y, et al Urbanization increases *Aedes albopictus* larval habitats and accelerates mosquito development and survivorship. PLoS Negl Trop Dis. 2014;8: e3301 10.1371/journal.pntd.0003301 25393814 PMC4230920

[3] Samson DM, Archer RS, Alimi TO, Arheart KL, Impoinvil DE, Oscar R, et al New baseline environmental assessment of mosquito ecology in northern Haiti during increased urbanization. J Vector Ecol. 2015;40: 46–58. 10.1111/jvec.12131 26047183 PMC4458708

[4] Urbinatti PR, Sendacz S, Natal D. Imaturos de mosquitos (Diptera: Culicidae) em parque de área metropolitana aberto à visitação pública. Rev Saude Publica. 2001;35: 461–466. 10.1590/S0034-89102001000500009 11723518

[5] Laporta GZ, Urbinatti PR, Natal D. Aspectos ecológicos da população de *Culex quinquefasciatus* Say (Diptera, Culicidae) em abrigos situados no Parque Ecológico do Tietê, São Paulo, SP. Rev Bras Entomol. 2006;50: 125–127. 10.1590/S0085-56262006000100019

[6] Ibañez-Justicia A, Cianci D. Modelling the spatial distribution of the nuisance mosquito species *Anopheles plumbeus* (Diptera: Culicidae) in the Netherlands. Parasit Vectors. 2015;8: 258 10.1186/s13071-015-0865-7 25927442 PMC4424539

[7] Medeiros-Sousa AR, Ceretti W, Urbinatti PR, de Carvalho GC, de Paula MB, Fernandes A, et al Mosquito fauna in municipal parks of São Paulo City, Brazil: a preliminary survey. J Am Mosq Control Assoc. 2013;29: 275–9. 10.2987/12-6304R.1 24199502

[8] Ceretti-junior W, Christe RDO, Rizzo M, Strobel C, Otavio M, Junior DM, et al Species composition and ecological aspects of immature mosquitoes (Diptera : Culicidae) in bromeliads in urban parks in the city of São Paulo. J Arthropod Borne Dis. 2016; 10(1): 102–112. 27047978 PMC4813398

[9] Ceretti-Júnior W, Medeiros-Sousa AR, Multini LC, Urbinatti PR, Vendrami DP, Natal D, et al Immature mosquitoes in bamboo internodes in municipal parks, city of São Paulo, Brazil. J Am Mosq Control Assoc. 2014;30: 268–274. 10.2987/14-6403R.1 25843132

[10] Forattini OP. Culicidologia Médica: Identificação, Biologia e Epidemiologia Culicidologia Médica. v.2. São Paulo: EDUSP; 2002.

[11] WRBU. Systematic Catalog of Culicidae. 2015. Available: http://www.mosquitocatalog.org/taxon_descr.aspx?ID=16082.

[12] Ceretti-Júnior W, Medeiros-Sousa AR, Bruno Wilke AB, Strobel RC, Dias Orico L, Souza Teixeira R, et al Mosquito faunal survey in a central park of the city of São Paulo, Brazil. J Am Mosq Control Assoc. 2015;31: 172–176. 10.2987/14-6457R 26181694

[13] de Carvalho GC, Malafronte RDS, Miti Izumisawa C, Souza Teixeira R, Natal L, Marrelli MT. Blood meal sources of mosquitoes captured in municipal parks in São Paulo, Brazil. J Vector Ecol. 2014;39: 146–52. 10.1111/j.1948-7134.2014.12081.x 24820567

[14] Davis C, Shannon R. Studies on yellow fever in South America: attempts to transmit the virus with certain Aedine and Sabethine mosquitoes and with *Triatomas* (Hemiptera). Am J Trop Med Hyg. 1931;11: 21–29.

[15] Kasai N. Susceptibilidade do mosquito Aedes fluviatilis (Lutz, 1904) à infecção por Dirofilaria immitis (Leidy, 1856). M.Sc. Thesis, Universidade Federal de Minas Gerais. 1979.

[16] de Camargo M V, Cônsoli RAGB, Williams P, Krettli AU, Camargo de MVT, Cônsoli RAGB, et al Factors influencing the development of *Plasmodium gallinaceum* in *Aedes fluviatilis*. Mem Inst Oswaldo Cruz. 1983;78: 83–94. 10.1590/S0074-02761983000100010 6645948

[17] Vezzani D, Eiras DF, Wisnivesky C. Dirofilariasis in Argentina: Historical review and first report of *Dirofilaria immitis* in a natural mosquito population. Vet Parasitol. 2006;136: 259–273. 10.1016/j.vetpar.2005.10.026 16310953

[18] Baton LA, Pacidônio EC, Gonçalves DDS, Moreira LA. w*Flu*: Characterization and evaluation of a native *Wolbachia* from the mosquito *Aedes fluviatilis* as a potential vector control agent. PLoS One. 2013;8 10.1371/journal.pone.0059619 PMC3608659 23555728

[19] Moreira L, Iturbe-Ormaetxe I, Jeffery J, Lu G, Pyke AT, Hedges LM, et al A *Wolbachia* symbiont in *Aedes aegypti* limits infection with Dengue, Chikungunya, and *Plasmodium*. Cell. 2009;139: 1268–1278. 10.1016/j.cell.2009.11.042 20064373

[20] Edman J. Disease control through manipulation of vector-host interaction: some historical and evolutionary perspectives. In: Proceedings of a Symposium: The Role of vector-host interactions in disease transmission. 1988.

[21] Descloux E, Mangeas M, Menkes CE, Lengaigne M, Leroy A, Tehei T, et al Climate-based models for understanding and forecasting dengue epidemics. Anyamba A, editor. PLoS Negl Trop Dis. 2012;6: e1470 10.1371/journal.pntd.0001470 22348154 PMC3279338

[22] Chaves LF, Koenraadt CJM. Climate change and highland malaria: fresh air for a hot debate. Q Rev Biol. 2010;85: 27–55. 10.1086/650284 20337259

[23] Brown JE, McBride CS, Johnson P, Ritchie S, Paupy C, Bossin H, et al Worldwide patterns of genetic differentiation imply multiple “domestications” of *Aedes aegypti*, a major vector of human diseases. Proc Biol Sci. 2011;278: 2446–2454. 10.1098/rspb.2010.2469 21227970 PMC3125627

[24] Edillo FE, Tripet F, McAbee RD, Foppa IM, Lanzaro GC, Cornel a J, et al A set of broadly applicable microsatellite markers for analyzing the structure of *Culex pipiens* (Diptera: Culicidae) populations. J Med Entomol. 2007;44: 145–149. 17294932 10.1603/0022-2585(2007)44[145:asobam]2.0.co;2

[25] Wilke A, Vidal P, Suesdek L, Marrelli M. Population genetics of neotropical *Culex quinquefasciatus* (Diptera: Culicidae). Parasit Vectors. 2014;7: 468 10.1186/s13071-014-0468-8 25280576 PMC4190383

[26] Fonseca DM, Smith JL, Wilkerson RC, Fleischer RC. Pathways of expansion and multiple introductions illustrated by large genetic differentiation among worldwide populations of the southern house mosquito. Am J Trop Med Hyg. 2006;74: 284–289. 16474085

[27] Olanratmanee P, Kittayapong P, Chansang C, Hoffmann A, Weeks AR, Endersby NM. Population genetic structure of *Aedes (Stegomyia) aegypti* (L.) at a micro-spatial scale in Thailand: implications for a dengue suppression strategy. PLoS Negl Trop Dis. 2013;7 10.1371/journal.pntd.0001913 PMC3542184 23326609

[28] Piccinali RV, Gürtler RE. Fine-scale genetic structure of *Triatoma infestans* in the Argentine Chaco. Infect Genet Evol. 2015;34: 143–152. 10.1016/j.meegid.2015.05.030 26027923

[29] Nasci RS. A lightweight battery-powered aspirator for collecting resting mosquitoes in the field. Mosq News 41: 808–811.

[30] Gomes A de C, Rabello EX, Natal D. Uma nova câmara coletora para armadilha CDC-miniatura. Rev Saude Publica. 1985;19: 190–191. 4089514 10.1590/s0034-89101985000200009

[31] Multini LC, Marrelli MT, Wilke ABB. Microsatellite loci cross-species transferability in *Aedes fluviatilis* (Diptera:Culicidae): a cost-effective approach for population genetics studies. Parasit Vectors. 2015;8: 635 10.1186/s13071-015-1256-9 26667177 PMC4678524

[32] Porretta D, Bellini R, Urbanelli S. Characterization of microsatellite markers in the mosquito *Ochlerotatus caspius* (Diptera: Culicidae). Mol Ecol Notes. 2005;5: 48–50. 10.1111/j.1471-8286.2004.00826.x

[33] Chambers EW, Meece JK, McGowan Ja, Lovin DD, Hemme RR, Chadee DD, et al Microsatellite isolation and linkage group identification in the yellow fever mosquito *Aedes aegypti*. J Hered. 2007;98: 202–210. 10.1093/jhered/esm015 17420178

[34] Beebe NW, Ambrose L, Hill La, Davis JB, Hapgood G, Cooper RD, et al Tracing the Tiger: Population genetics provides valuable insights into the *Aedes (Stegomyia) albopictus* invasion of the Australasian region. PLoS Negl Trop Dis. 2013;7 10.1371/journal.pntd.0002361 PMC3738475 23951380

[35] Rousset F. genepop’007: a complete re-implementation of the genepop software for Windows and Linux. Mol Ecol Resour. 2008;8: 103–106. 10.1111/j.1471-8286.2007.01931.x 21585727

[36] Excoffier L, Laval G, Schneider S. Arlequin (version 3.0): An integrated software package for population genetics data analysis. Evol Bioinform Online. 2005;1: 47–50. PMC2658868 19325852

[37] Kalinowski ST. hp-rare 1.0: a computer program for performing rarefaction on measures of allelic richness. Mol Ecol Notes. 2005;5: 187–189. 10.1111/j.1471-8286.2004.00845.x

[38] Chapuis MP, Estoup A. Microsatellite null alleles and estimation of population differentiation. Mol Biol Evol. 2006;24: 621–631. 10.1093/molbev/msl191 17150975

[39] Patton D. A diversity index for quantifying habitat "edge”. Wildl Soc Bull. 1975;3: 171–173.

[40] McGaughran A, Morgan K, Sommer RJ. Environmental variables explain genetic structure in a beetle-associated nematode. PLoS One. 2014;9: e87317 10.1371/journal.pone.0087317 24498073 PMC3909076

[41] Hammer Ø, Harper DATT, Ryan PD. PAST: Paleontological Statistics Software Package for Education and Data Analysis. Palaeontol Electron. 2001;4: 9.

[42] StatSoft I. STATISTICA (data analysis software system), version 7. 2004. Available: www.statsoft.com

[43] Pritchard JK, Stephens M, Donnelly P. Inference of population structure using multilocus genotype data: dominant markers and null alleles. Mol Ecol Notes. 2007;7: 574–578. 10.1111/j.1471-8286.2007.01758.x 18784791 PMC1974779

[44] Evanno G, Regnaut S, Goudet J. Detecting the number of clusters of individuals using the software structure: a simulation study. Mol Ecol. 2005;14: 2611–2620. 10.1111/j.1365-294X.2005.02553.x 15969739

[45] Cornuet JM, Luikart G. Description and power analysis of two tests for detecting recent population bottlenecks from allele frequency data. Genetics. 1996;144: 2001–2014. 8978083 10.1093/genetics/144.4.2001 PMC1207747

[46] McKinney ML. Effects of urbanization on species richness: A review of plants and animals. Urban Ecosyst. 2008;11: 161–176. 10.1007/s11252-007-0045-4

[47] Pascual M, Ahumada JA, Chaves LF, Rodo X, Bouma M. Malaria resurgence in the East African highlands: Temperature trends revisited. Proc Natl Acad Sci. 2006;103: 5829–5834. 10.1073/pnas.0508929103 16571662 PMC1416896

[48] Donnelly MJ, Licht MC, Lehmann T. Evidence for recent population expansion in the evolutionary history of the malaria vectors *Anopheles arabiensis* and *Anopheles gambiae*. Mol Biol Evol. 2001;18: 1353–1364. 10.1093/oxfordjournals.molbev.a003919 11420373

[49] Michel AP. Divergence with gene flow in *Anopheles funestus* from the sudan savanna of Burkina Faso, West Africa. Genetics. 2006;173: 1389–1395. 10.1534/genetics.106.059667 16648581 PMC1526678

[50] Mirabello L, Conn JE. Molecular population genetics of the malaria vector *Anopheles darlingi* in central and South America. Heredity. 2006;97: 438–438. 10.1038/sj.hdy.6800913 16508661

[51] LaDeau SL, Allan BF, Leisnham PT, Levy MZ. The ecological foundations of transmission potential and vector-borne disease in urban landscapes. Funct Ecol. 2015;29: 889–901. 10.1111/1365-2435.12487 26549921 PMC4631442

[52] Bello FJ, Segura NA, Ruiz-García M. Analysis of the genetic variability and structure of *Ochlerotatus taeniorhynchus* (Diptera: Culicidae) populations from the Colombian Atlantic coast on the basis of random amplified polymorphic DNA markers. Genet Mol Res. 2014;13: 4110–4123. 10.4238/2014.May.30.6 24938703

[53] Bello F, Becerra V. Genetic variability and heterogeneity of Venezuelan equine encephalitis virus vector *Ochlerotatus taeniorhynchus* (Diptera: Culicidae) populations of the Colombian Atlantic coast, based on microsatellite loci. Genet Mol Res. 2009;8: 1179–1190. 10.4238/vol8-3gmr652 19866436

[54] Tauil PL. Urbanization and dengue ecology. Cad saude publica. 2001;17: 99–102. S0102-311X2001000700018 11426270

[55] Brown JE, Evans BR, Zheng W, Obas V, Barrera-Martinez L, Egizi A, et al Human impacts have shaped historical and recent evolution in *Aedes aegypti*, the dengue and yellow fever mosquito. Evolution. 2014;68: 514–525. 10.1111/evo.12281 24111703 PMC3946797

[56] Zielke DE, Werner D, Schaffner F, Kampen H, Fonseca DM. Unexpected patterns of admixture in German populations of *Aedes japonicus japonicus* (Diptera: Culicidae) underscore the importance of human intervention. PLoS One. 2014;9: e99093 10.1371/journal.pone.0099093 24992470 PMC4081119

[57] Paaijmans KP, Heinig RL, Seliga R a., Blanford JI, Blanford S, Murdock CC, et al Temperature variation makes ectotherms more sensitive to climate change. Glob Chang Biol. 2013;19: 2373–2380. 10.1111/gcb.12240 23630036 PMC3908367

[58] Levy MZ, Barbu CM, Castillo-Neyra R, Quispe-Machaca VR, Ancca-Juarez J, Escalante-Mejia P, et al Urbanization, land tenure security and vector-borne Chagas disease. Proc R Soc B Biol Sci. 2014;281: 20141003–20141003. 10.1098/rspb.2014.1003 PMC4100517 24990681

[59] Pudovkin I, Zaykin DV, Hedgecock D. On the potential for estimating the effective number of breeders from heterozygote-excess in progeny. Genetics. 1996;144: 383–387. 8878701 10.1093/genetics/144.1.383 PMC1207510

[60] Monteiro F, Shama R, Martins AJ, Gloria-Soria A, Brown JE, Powell JR. Genetic diversity of Brazilian *Aedes aegypti*: Patterns following an eradication program. PLoS Negl Trop Dis. 2014;8: e3167 10.1371/journal.pntd.0003167 25233218 PMC4169244

[61] Louise C, Vidal PO, Suesdek L. Microevolution of *Aedes aegypti*. PLoS One. 2015;10: e0137851 10.1371/journal.pone.0137851 26360876 PMC4567268

[62] Carlsson J. Effects of microsatellite null alleles on assignment testing. J Hered. 2008;99: 616–623. 10.1093/jhered/esn048 18535000

[63] Belisário CJ, Pessoa GCD, dos Santos PF, Dias LS, Rosa ACL, Diotaiuti L. Markers for the population genetics studies of *Triatoma sordida* (Hemiptera: Reduviidae). Parasit Vectors. 2015;8: 269 10.1186/s13071-015-0879-1 25963633 PMC4435924

[64] Zanluca C, Melo VCA de, Mosimann ALP, Santos GIV dos, Santos CND dos, Luz K. First report of autochthonous transmission of Zika virus in Brazil. Mem Inst Oswaldo Cruz. 2015;110: 569–572. 10.1590/0074-02760150192 26061233 PMC4501423

[65] Honório NA, Câmara DCP, Calvet GA, Brasil P. Chikungunya: uma arbovirose em estabelecimento e expansão no Brasil. Cad Saude Publica. 2015;31: 906–908. 10.1590/0102-311XPE020515 26083166

[66] Venkatesan M, Rasgon JL. Mosquito-mediated dispersal of West Nile Virus. Mol Ecol. 2012;19: 1573–1584. 10.1111/j.1365-294X.2010.04577.x PMC3253699 20298466

[67] Tchouassi DP, Bastos ADS, Sole CL, Diallo M, Lutomiah J, Mutisya J, et al Population genetics of two key mosquito vectors of Rift Valley Fever Virus reveals new insights into the changing disease outbreak patterns in Kenya. PLoS Negl Trop Dis. 2014;8: e3364 10.1371/journal.pntd.0003364 25474018 PMC4256213

