# Supplementary figures and images for: Population Genetic Structure of Aedes fluviatilis (Diptera: Culicidae)
Source: PLoS One. 2016 Sep 6;11(9):e0162328. doi: 10.1371/journal.pone.0162328 (PMC5012556; doi:10.1371/journal.pone.0162328)

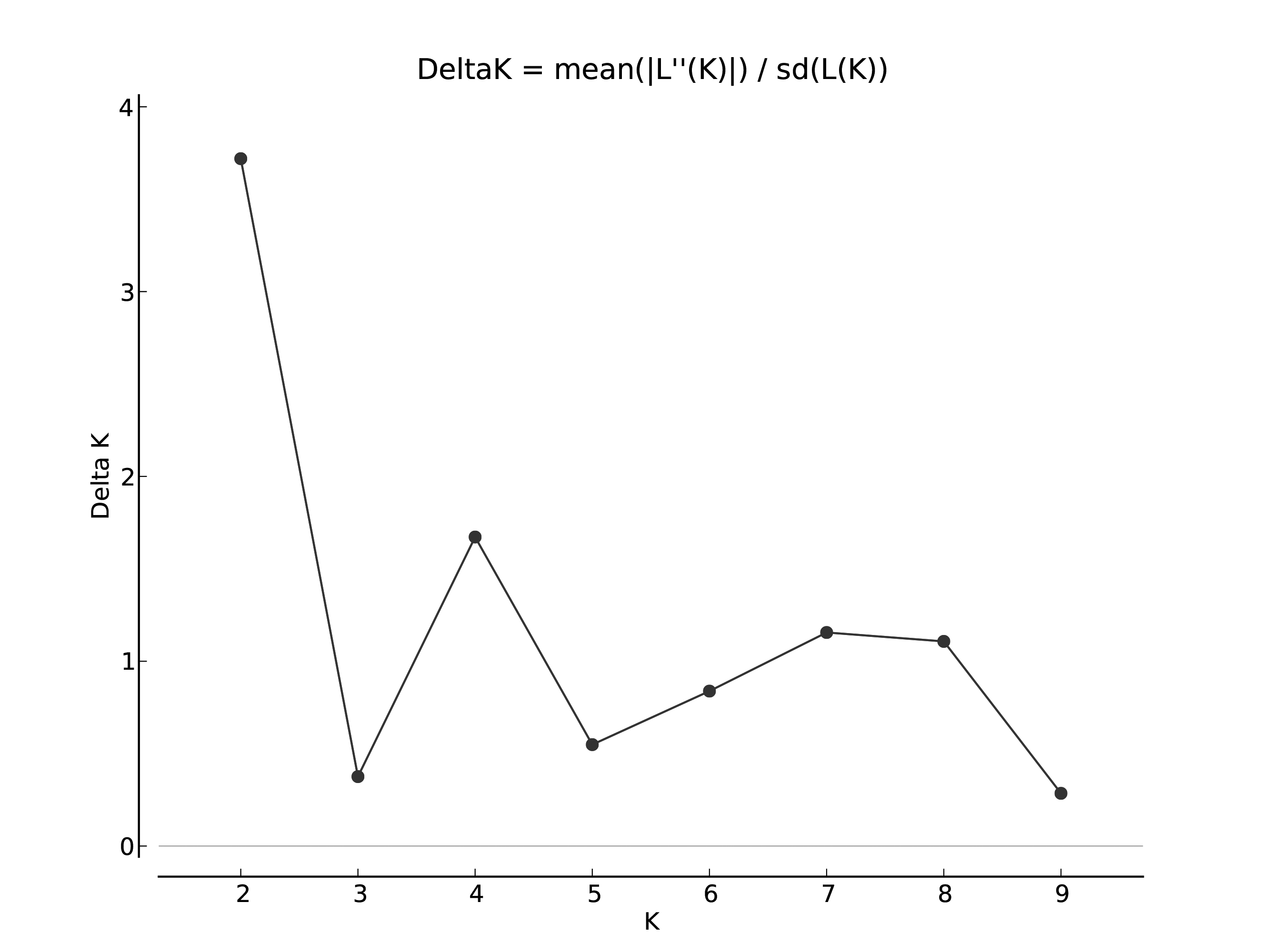

Supplement: S1 Fig — (TIF) [file pone.0162328.s001.tif]
