# Supplementary material for: Population Genetic Structure of Aedes fluviatilis (Diptera: Culicidae)
Source: PLoS One. 2016 Sep 6;11(9):e0162328. doi: 10.1371/journal.pone.0162328 (PMC5012556; doi:10.1371/journal.pone.0162328)
Supplement: S1 Table — Significant P-values in bold. (DOCX) [file pone.0162328.s002.docx]

**S1 Table.** Analysis of the genetic diversity of *Aedes fluviatilis* using eight microsatellite loci.

| **Population** | **Locus** | **Number of alleles** | **Observed Heterozygosity** | **Expected Heterozygosity** | ***Fis*** | ***P*** |
| --- | --- | --- | --- | --- | --- | --- |
| Burle Marx | OchcB5 | 1 | / | / | / | / |
|  | OchcB9 | 5 | 0.233 | 0.481 | 0.5201 | 0.00723 |
|  | OchcD11 | 3 | 0.933 | 0.521 | -0.8145 | **<0.00001** |
|  | Albtri3 | 13 | 0.586 | 0.860 | 0.3224 | **<0.00001** |
|  | Albtri33 | 1 | / | / | / | / |
|  | Albtri20 | 5 | 0.100 | 0.129 | 0.2335 | 0.04396 |
|  | AEDC | 2 | 0.933 | 0.506 | -0.8710 | **<0.00001** |
|  | Albtri44 | 5 | 0.966 | 0.662 | -0.4716 | **<0.00001** |
|  |  |  |  |  |  |  |
| Ibirapuera | OchcB5 | 2 | 0 | 0.363 | 1 | **<0.00001** |
|  | OchcB9 | 3 | 0.333 | 0.371 | 0.1036 | 0.68683 |
|  | OchcD11 | 4 | 0.966 | 0.571 | -0.7128 | **<0.00001** |
|  | Albtri3 | 7 | 0.793 | 0.799 | 0.0085 | **<0.00001** |
|  | Albtri33 | 3 | 0.033 | 0.157 | 0.7914 | 0.00168 |
|  | Albtri20 | 1 | / | / | / | / |
|  | AEDC | 2 | 1 | 0.508 | -1 | **<0.00001** |
|  | Albtri44 | 6 | 0.966 | 0.624 | -0.5617 | **<0.00001** |
|  |  |  |  |  |  |  |
| Piquerí | OchcB5 | 2 | 0 | 0.235 | 1 | **<0.00001** |
|  | OchcB9 | 3 | 0.333 | 0.371 | 0.1036 | 0.68970 |
|  | OchcD11 | 4 | 0.933 | 0.597 | -0.5767 | **<0.00001** |
|  | Albtri3 | 11 | 0.923 | 0.766 | -0.2097 | 0.06812 |
|  | Albtri33 | 3 | 0.100 | 0.097 | -0.0235 | 1 |
|  | Albtri20 | 2 | 0.035 | 0.035 | / | 1 |
|  | AEDC | 3 | 1 | 0.524 | -0.9355 | **<0.00001** |
|  | Albtri44 | 6 | 1 | 0.670 | -0.5052 | **<0.00001** |
|  |  |  |  |  |  |  |
| Previdência | OchcB5 | 1 | / | / | / | / |
|  | OchcB9 | 2 | 0.300 | 0.304 | 0.0151 | 1 |
|  | OchcD11 | 3 | 0.866 | 0.569 | -0.5356 | **<0.00001** |
|  | Albtri3 | 9 | 0.689 | 0.827 | 0.1691 | **<0.00001** |
|  | Albtri33 | 2 | 0.133 | 0.183 | 0.2750 | 0.23455 |
|  | Albtri20 | 3 | 0.066 | 0.066 | -0.0087 | 1 |
|  | AEDC | 2 | 1 | 0.508 | -1 | **<0.00001** |
|  | Albtri44 | 7 | 0.966 | 0.726 | -0.3381 | **<0.00001** |
|  |  |  |  |  |  |  |
| Santo Dias | OchcB5 | 2 | 0 | 0.065 | 1 | 0.01376 |
|  | OchcB9 | 2 | 0.233 | 0.304 | 0.2368 | 0.23673 |
|  | OchcD11 | 3 | 0.966 | 0.575 | -0.6990 | **<0.00001** |
|  | Albtri3 | 10 | 0.700 | 0.837 | 0.1663 | **<0.00001** |
|  | Albtri33 | 2 | 0.433 | 0.345 | -0.2609 | 0.29782 |
|  | Albtri20 | 6 | 0.266 | 0.299 | 0.1111 | **<0.00001** |
|  | AEDC | 2 | 1 | 0.508 | -1 | **<0.00001** |
|  | Albtri44 | 10 | 0.966 | 0.768 | -0.2628 | **<0.00001** |
|  |  |  |  |  |  |  |
| Shangrilá | OchcB5 | 1 | / | / | / | / |
|  | OchcB9 | 2 | 0.366 | 0.462 | 0.2104 | 0.41099 |
|  | OchcD11 | 4 | 0.933 | 0.620 | -0.5178 | **<0.00001** |
|  | Albtri3 | 13 | 0.931 | 0.850 | -0.0964 | 0.00624 |
|  | Albtri33 | 2 | 0.366 | 0.304 | -0.2083 | 0.54535 |
|  | Albtri20 | 3 | 0.166 | 0.157 | -0.0584 | 1 |
|  | AEDC | 2 | 0.966 | 0.507 | -0.9333 | **<0.00001** |
|  | Albtri44 | 5 | 1 | 0.707 | -0.4227 | **<0.00001** |
|  |  |  |  |  |  |  |
| Alfredo Volpi | OchcB5 | 2 | 0 | 0.067 | 1 | 0.01921 |
|  | OchcB9 | 3 | 0.344 | 0.346 | 0.0036 | 0.01663 |
|  | OchcD11 | 3 | 0.896 | 0.539 | -0.6813 | **<0.00001** |
|  | Albtri3 | 15 | 0.551 | 0.860 | 0.3632 | **<0.00001** |
|  | Albtri33 | 1 | / | / | / | / |
|  | Albtri20 | 3 | 0.103 | 0.101 | -0.0244 | 1 |
|  | AEDC | 2 | 0.892 | 0.503 | -0.8000 | **<0.00001** |
|  | Albtri44 | 5 | 0.730 | 0.608 | -0.2056 | 0.02287 |
| Chico Mendes | OchcB5 | 1 | / | / | / | / |
|  | OchcB9 | 2 | 0.166 | 0.304 | 0.4569 | 0.03644 |
|  | OchcD11 | 3 | 0.900 | 0.592 | -0.5323 | **<0.00001** |
|  | Albtri3 | 13 | 0.500 | 0.867 | 0.4276 | **<0.00001** |
|  | Albtri33 | 2 | 0.033 | 0.033 | / | 1 |
|  | Albtri20 | 3 | 0.100 | 0.097 | -0.0235 | 1 |
|  | AEDC | 2 | 0.900 | 0.503 | -0.8125 | **<0.00001** |
|  | Albtri44 | 6 | 0.733 | 0.695 | -0.0554 | **<0.00001** |
| Carmo | OchcB5 | 1 | / | / | / | / |
|  | OchcB9 | 3 | 0.413 | 0.387 | -0.0701 | 1 |
|  | OchcD11 | 2 | 0.896 | 0.503 | -0.8065 | **<0.00001** |
|  | Albtri3 | 14 | 0.518 | 0.857 | 0.3998 | **<0.00001** |
|  | Albtri33 | 3 | 0.133 | 0.128 | -0.0404 | 1 |
|  | Albtri20 | 5 | 0.166 | 0.160 | -0.0394 | 1 |
|  | AEDC | 2 | 0.931 | 0.506 | -0.8667 | **<0.00001** |
|  | Albtri44 | 9 | 0.655 | 0.759 | 0.1392 | 0.01842 |

Significant *P*-values in bold.
