# Supplementary material for: Population Genetic Structure of Aedes fluviatilis (Diptera: Culicidae)
Source: PLoS One. 2016 Sep 6;11(9):e0162328. doi: 10.1371/journal.pone.0162328 (PMC5012556; doi:10.1371/journal.pone.0162328)
Supplement: S2 Table — Allelic richness (Na) and private allelic richness (Np). (DOCX) [file pone.0162328.s003.docx]

**S2** **Table.** Allele frequencies for the eight loci analyzed in *Aedes fluviatilis* populations.

| **Locus** | **Allele** | **Burle Marx** | **Ibirapuera** | **Piquerí** | **Previdência** | **Santo Dias** | **Shangrilá** | **Alfredo Volpi** | **Chico Mendes** | **Carmo** |
| --- | --- | --- | --- | --- | --- | --- | --- | --- | --- | --- |
| **OchcB5** | 194 | 0.000 | 0.2333 | 0.1333 | 0.000 | 0.0333 | 0.000 | 0.0345 | 0.000 | 0.000 |
|  | 196 | 1.000 | 0.7667 | 0.8667 | 1.000 | 0.9667 | 1.000 | 0.9655 | 1.000 | 1.000 |
| **OchcB9** | 90 | 0.000 | 0.000 | 0.000 | 0.000 | 0.000 | 0.000 | 0.0345 | 0.000 | 0.000 |
|  | 280 | 0.1667 | 0.2167 | 0.2167 | 0.1833 | 0.1833 | 0.3500 | 0.1724 | 0.1833 | 0.2069 |
|  | 282 | 0.0333 | 0.000 | 0.0167 | 0.000 | 0.000 | 0.000 | 0.000 | 0.000 | 0.0345 |
|  | 284 | 0.0167 | 0.0167 | 0.000 | 0.000 | 0.000 | 0.000 | 0.000 | 0.000 | 0.000 |
|  | 286 | 0.7000 | 0.7667 | 0.7667 | 0.8167 | 0.8167 | 0.6500 | 0.7931 | 0.8167 | 0.7586 |
|  | 288 | 0.0833 | 0.000 | 0.000 | 0.000 | 0.000 | 0.000 | 0.000 | 0.000 | 0.000 |
| **OchcD11** | 88 | 0.0167 | 0.0167 | 0.0167 | 0.000 | 0.000 | 0.0167 | 0.0345 | 0.000 | 0.000 |
|  | 90 | 0.4500 | 0.4667 | 0.4500 | 0.4667 | 0.5167 | 0.4500 | 0.5172 | 0.4833 | 0.4483 |
|  | 130 | 0.000 | 0.0500 | 0.0833 | 0.0667 | 0.0833 | 0.1167 | 0.000 | 0.1000 | 0.000 |
|  | 132 | 0.5333 | 0.4667 | 0.4500 | 0.4667 | 0.4000 | 0.4167 | 0.4483 | 0.4167 | 0.5517 |
| **Albtri3** | 93 | 0.000 | 0.000 | 0.000 | 0.000 | 0.000 | 0.000 | 0.0172 | 0.000 | 0.000 |
|  | 108 | 0.000 | 0.000 | 0.000 | 0.000 | 0.000 | 0.000 | 0.000 | 0.000 | 0.0370 |
|  | 113 | 0.000 | 0.000 | 0.000 | 0.000 | 0.000 | 0.000 | 0.000 | 0.0167 | 0.000 |
|  | 117 | 0.000 | 0.000 | 0.000 | 0.000 | 0.000 | 0.000 | 0.000 | 0.000 | 0.0185 |
|  | 120 | 0.000 | 0.000 | 0.000 | 0.000 | 0.000 | 0.000 | 0.000 | 0.000 | 0.0185 |
|  | 126 | 0.000 | 0.000 | 0.000 | 0.000 | 0.000 | 0.000 | 0.000 | 0.0167 | 0.000 |
|  | 141 | 0.000 | 0.000 | 0.000 | 0.000 | 0.000 | 0.000 | 0.0172 | 0.0167 | 0.0185 |
|  | 150 | 0.000 | 0.000 | 0.000 | 0.000 | 0.000 | 0.000 | 0.0172 | 0.000 | 0.000 |
|  | 168 | 0.000 | 0.000 | 0.000 | 0.0172 | 0.000 | 0.000 | 0.0172 | 0.000 | 0.000 |
|  | 173 | 0.000 | 0.000 | 0.0192 | 0.000 | 0.000 | 0.000 | 0.000 | 0.000 | 0.000 |
|  | 183 | 0.000 | 0.000 | 0.000 | 0.000 | 0.000 | 0.000 | 0.0172 | 0.000 | 0.000 |
|  | 186 | 0.000 | 0.000 | 0.000 | 0.000 | 0.000 | 0.000 | 0.000 | 0.000 | 0.000 |
|  | 189 | 0.000 | 0.000 | 0.0192 | 0.000 | 0.000 | 0.000 | 0.000 | 0.000 | 0.000 |
|  | 195 | 0.000 | 0.000 | 0.000 | 0.0172 | 0.000 | 0.000 | 0.000 | 0.000 | 0.000 |
|  | 201 | 0.000 | 0.000 | 0.000 | 0.000 | 0.000 | 0.000 | 0.000 | 0.000 | 0.0185 |
|  | 204 | 0.000 | 0.000 | 0.000 | 0.000 | 0.000 | 0.000 | 0.000 | 0.000 | 0.0185 |
|  | 207 | 0.0172 | 0.000 | 0.000 | 0.000 | 0.000 | 0.000 | 0.000 | 0.000 | 0.000 |
|  | 261 | 0.000 | 0.000 | 0.000 | 0.000 | 0.000 | 0.000 | 0.000 | 0.0167 | 0.000 |
|  | 297 | 0.000 | 0.000 | 0.0192 | 0.000 | 0.000 | 0.000 | 0.000 | 0.000 | 0.000 |
|  | 312 | 0.000 | 0.000 | 0.000 | 0.000 | 0.000 | 0.000 | 0.000 | 0.0167 | 0.000 |
|  | 321 | 0.0172 | 0.000 | 0.000 | 0.000 | 0.000 | 0.000 | 0.000 | 0.000 | 0.000 |
|  | 330 | 0.000 | 0.000 | 0.000 | 0.000 | 0.000 | 0.000 | 0.0172 | 0.000 | 0.000 |
|  | 336 | 0.000 | 0.000 | 0.000 | 0.000 | 0.000 | 0.0172 | 0.000 | 0.000 | 0.000 |
|  | 342 | 0.0172 | 0.000 | 0.000 | 0.000 | 0.000 | 0.000 | 0.0172 | 0.000 | 0.000 |
|  | 345 | 0.0862 | 0.000 | 0.000 | 0.000 | 0.000 | 0.0345 | 0.000 | 0.000 | 0.000 |
|  | 354 | 0.000 | 0.000 | 0.0192 | 0.000 | 0.000 | 0.000 | 0.000 | 0.000 | 0.000 |
|  | 360 | 0.000 | 0.000 | 0.000 | 0.000 | 0.0167 | 0.000 | 0.000 | 0.000 | 0.0185 |
|  | 363 | 0.0172 | 0.000 | 0.000 | 0.000 | 0.0167 | 0.0172 | 0.000 | 0.000 | 0.000 |
|  | 369 | 0.000 | 0.0172 | 0.000 | 0.000 | 0.000 | 0.0517 | 0.000 | 0.000 | 0.000 |
|  | 372 | 0.0517 | 0.3621 | 0.4423 | 0.2931 | 0.2167 | 0.3276 | 0.2759 | 0.1333 | 0.1852 |
|  | 375 | 0.0690 | 0.0862 | 0.0192 | 0.1034 | 0.1167 | 0.0862 | 0.0172 | 0.0167 | 0.0370 |
|  | 381 | 0.000 | 0.000 | 0.000 | 0.0345 | 0.000 | 0.0172 | 0.000 | 0.000 | 0.000 |
|  | 396 | 0.000 | 0.000 | 0.000 | 0.000 | 0.000 | 0.000 | 0.000 | 0.0167 | 0.000 |
|  | 405 | 0.0345 | 0.1034 | 0.0577 | 0.000 | 0.1167 | 0.0345 | 0.0517 | 0.1000 | 0.0556 |
|  | 408 | 0.0345 | 0.000 | 0.000 | 0.0172 | 0.0333 | 0.0690 | 0.1207 | 0.0333 | 0.0185 |
|  | 411 | 0.2069 | 0.1034 | 0.1538 | 0.1552 | 0.3500 | 0.1207 | 0.0862 | 0.2833 | 0.2222 |
|  | 414 | 0.2069 | 0.1724 | 0.1154 | 0.1552 | 0.0833 | 0.0690 | 0.1897 | 0.1667 | 0.2407 |
|  | 417 | 0.2241 | 0.1552 | 0.0962 | 0.2069 | 0.1167 | 0.1379 | 0.1207 | 0.1167 | 0.0926 |
|  | 429 | 0.0172 | 0.000 | 0.000 | 0.000 | 0.000 | 0.000 | 0.000 | 0.000 | 0.000 |
|  | 450 | 0.000 | 0.000 | 0.0385 | 0.000 | 0.000 | 0.000 | 0.000 | 0.000 | 0.000 |
|  | 453 | 0.000 | 0.000 | 0.000 | 0.000 | 0.0167 | 0.0172 | 0.000 | 0.000 | 0.000 |
| **Albtri33** | 105 | 0.000 | 0.000 | 0.000 | 0.000 | 0.000 | 0.000 | 0.000 | 0.0167 | 0.000 |
|  | 108 | 0.000 | 0.0667 | 0.0333 | 0.000 | 0.000 | 0.000 | 0.000 | 0.000 | 0.000 |
|  | 111 | 0.000 | 0.000 | 0.000 | 0.000 | 0.000 | 0.000 | 0.000 | 0.000 | 0.0167 |
|  | 113 | 0.000 | 0.0167 | 0.000 | 0.1000 | 0.2167 | 0.1833 | 0.000 | 0.000 | 0.0500 |
|  | 117 | 0.000 | 0.000 | 0.0167 | 0.000 | 0.000 | 0.000 | 0.000 | 0.000 | 0.000 |
|  | 120 | 1.000 | 0.9167 | 0.9500 | 0.9000 | 0.7833 | 0.8167 | 1.000 | 0.9833 | 0.9333 |
| **Albtri20** | 102 | 0.000 | 0.000 | 0.000 | 0.0167 | 0.0167 | 0.000 | 0.000 | 0.000 | 0.000 |
|  | 117 | 0.000 | 0.000 | 0.000 | 0.000 | 0.000 | 0.000 | 0.000 | 0.0167 | 0.000 |
|  | 119 | 0.000 | 0.000 | 0.000 | 0.000 | 0.000 | 0.000 | 0.000 | 0.0333 | 0.000 |
|  | 120 | 0.000 | 0.000 | 0.000 | 0.0167 | 0.1000 | 0.0667 | 0.0345 | 0.000 | 0.0333 |
|  | 122 | 0.000 | 0.000 | 0.000 | 0.000 | 0.000 | 0.0167 | 0.000 | 0.000 | 0.000 |
|  | 135 | 0.000 | 0.000 | 0.0179 | 0.000 | 0.000 | 0.000 | 0.000 | 0.000 | 0.000 |
|  | 141 | 0.0167 | 0.000 | 0.000 | 0.000 | 0.000 | 0.000 | 0.000 | 0.000 | 0.000 |
|  | 144 | 0.000 | 0.000 | 0.000 | 0.000 | 0.000 | 0.000 | 0.000 | 0.000 | 0.0167 |
|  | 147 | 0.000 | 0.000 | 0.000 | 0.000 | 0.000 | 0.000 | 0.0172 | 0.000 | 0.0167 |
|  | 159 | 0.0167 | 0.000 | 0.000 | 0.000 | 0.0167 | 0.000 | 0.000 | 0.000 | 0.000 |
|  | 160 | 0.0167 | 0.000 | 0.000 | 0.000 | 0.000 | 0.000 | 0.000 | 0.000 | 0.000 |
|  | 162 | 0.9333 | 1.000 | 0.9821 | 0.9667 | 0.8333 | 0.9167 | 0.9483 | 0.9500 | 0.9334 |
|  | 165 | 0.000 | 0.000 | 0.000 | 0.000 | 0.0167 | 0.000 | 0.000 | 0.000 | 0.000 |
|  | 186 | 0.0167 | 0.000 | 0.000 | 0.000 | 0.000 | 0.000 | 0.000 | 0.000 | 0.000 |
|  | 219 | 0.000 | 0.000 | 0.000 | 0.000 | 0.0167 | 0.000 | 0.000 | 0.000 | 0.000 |
| **AEDC** | 165 | 0.000 | 0.000 | 0.0167 | 0.000 | 0.000 | 0.000 | 0.000 | 0.000 | 0.000 |
|  | 174 | 0.5333 | 0.5000 | 0.4833 | 0.5000 | 0.5000 | 0.5167 | 0.5536 | 0.5500 | 0.5345 |
|  | 180 | 0.4667 | 0.5000 | 0.5000 | 0.5000 | 0.5000 | 0.4833 | 0.4464 | 0.4500 | 0.4655 |
| **Albtri44** | 246 | 0.000 | 0.000 | 0.000 | 0.000 | 0.0167 | 0.000 | 0.000 | 0.0167 | 0.000 |
|  | 252 | 0.000 | 0.000 | 0.000 | 0.000 | 0.0500 | 0.000 | 0.0385 | 0.000 | 0.000 |
|  | 291 | 0.000 | 0.000 | 0.000 | 0.000 | 0.000 | 0.000 | 0.000 | 0.000 | 0.0172 |
|  | 294 | 0.4833 | 0.5167 | 0.4500 | 0.3833 | 0.3500 | 0.3833 | 0.2885 | 0.3667 | 0.3276 |
|  | 297 | 0.0333 | 0.000 | 0.000 | 0.0500 | 0.0500 | 0.1167 | 0.0769 | 0.0500 | 0.0517 |
|  | 303 | 0.000 | 0.000 | 0.000 | 0.000 | 0.0167 | 0.000 | 0.000 | 0.000 | 0.000 |
|  | 306 | 0.000 | 0.000 | 0.0333 | 0.0333 | 0.0333 | 0.000 | 0.000 | 0.000 | 0.0172 |
|  | 318 | 0.000 | 0.000 | 0.000 | 0.0167 | 0.000 | 0.000 | 0.000 | 0.000 | 0.000 |
|  | 342 | 0.000 | 0.000 | 0.0167 | 0.000 | 0.000 | 0.000 | 0.000 | 0.000 | 0.000 |
|  | 369 | 0.000 | 0.000 | 0.000 | 0.000 | 0.000 | 0.000 | 0.000 | 0.000 | 0.0172 |
|  | 447 | 0.0833 | 0.0667 | 0.1167 | 0.1500 | 0.1167 | 0.0667 | 0.0385 | 0.1333 | 0.1379 |
|  | 448 | 0.000 | 0.000 | 0.000 | 0.000 | 0.0167 | 0.000 | 0.000 | 0.000 | 0.000 |
|  | 450 | 0.0833 | 0.0500 | 0.0333 | 0.0333 | 0.000 | 0.0667 | 0.000 | 0.0333 | 0.0690 |
|  | 459 | 0.3167 | 0.3333 | 0.3500 | 0.3333 | 0.334 | 0.3667 | 0.5577 | 0.4000 | 0.3448 |
|  | 462 | 0.000 | 0.0167 | 0.000 | 0.000 | 0.000 | 0.000 | 0.000 | 0.000 | 0.000 |
|  | 477 | 0.000 | 0.0167 | 0.000 | 0.000 | 0.0167 | 0.000 | 0.000 | 0.000 | 0.0172 |
|  |  |  |  |  |  |  |  |  |  |  |
| ***Na*** |  | 4.2 | 3.4 | 4.15 | 3.53 | 4.43 | 3.91 | 4.12 | 3.84 | 4.72 |
| ***Np*** |  | 0.83 | 0.13 | 1.07 | 0.26 | 0.49 | 0.25 | 0.72 | 0.91 | 1.19 |

Allelic richness (Na) and private allelic richness (Np).
