# Supplementary material for: Population Genetic Structure of Aedes fluviatilis (Diptera: Culicidae)
Source: PLoS One. 2016 Sep 6;11(9):e0162328. doi: 10.1371/journal.pone.0162328 (PMC5012556; doi:10.1371/journal.pone.0162328)
Supplement: S3 Table — *Significant P-value. (DOCX) [file pone.0162328.s004.docx]

**S3 Table.** Linear correlation analysis of genetic distance [F_ST_/(1-F_ST_)] and environmental variables.

| F_ST_/(1-F_ST_) | R | r² | *P* |
| --- | --- | --- | --- |
| Elevation | -0.51321 | 0.26338 | 0.15765 |
| Patton index | -0.43596 | 0.19006 | 0.24078 |
| Green area per inhabitant (m²) | -0.51414 | 0.26434 | 0.15677 |
| Slope | -0.6694 | 0.4481 | **0.048595*** |
| Rainfall | -0.025753 | 0.0006632 | 0.94757 |
| Mean annual temperature | -0.2338 | 0.054662 | 0.54487 |
| Park area (km²) | 0.45157 | 0.20392 | 0.2224 |

*Significant *P*-value.
